# The Telomeric Protein TRF2 Binds the ATM Kinase and Can Inhibit the ATM-Dependent DNA Damage Response

**DOI:** 10.1371/journal.pbio.0020240

**Published:** 2004-08-17

**Authors:** Jan Karlseder, Kristina Hoke, Olga K Mirzoeva, Christopher Bakkenist, Michael B Kastan, John H. J Petrini, Titia de Lange

**Affiliations:** **1**Laboratory for Cell Biology and Genetics, Rockefeller University, New YorkUnited States of America; **2**Department of Hematology and Oncology, St. Jude Children's Research HospitalMemphis, Tennessee, United States of America; **3**Memorial Sloan–Kettering Cancer Center, New YorkNew YorkUnited States of America

## Abstract

The telomeric protein TRF2 is required to prevent mammalian telomeres from activating DNA damage checkpoints. Here we show that overexpression of TRF2 affects the response of the ATM kinase to DNA damage. Overexpression of TRF2 abrogated the cell cycle arrest after ionizing radiation and diminished several other readouts of the DNA damage response, including phosphorylation of Nbs1, induction of p53, and upregulation of p53 targets. TRF2 inhibited autophosphorylation of ATM on S1981, an early step in the activation of this kinase. A region of ATM containing S1981 was found to directly interact with TRF2 in vitro, and ATM immunoprecipitates contained TRF2. We propose that TRF2 has the ability to inhibit ATM activation at telomeres. Because TRF2 is abundant at chromosome ends but not elsewhere in the nucleus, this mechanism of checkpoint control could specifically block a DNA damage response at telomeres without affecting the surveillance of chromosome internal damage.

## Abstract 2

The human telomere-associated protein TRF2 inhibits ATM kinase, suggesting a mechanism by which the telomeric protein complex prevents the activation of this DNA damage response transducer

## Introduction

Telomeres prevent the recognition of natural chromosome ends as double-stranded breaks (DSBs). When telomeres become dysfunctional due to shortening or loss of protective factors, chromosome ends activate a DNA damage response mediated (in part) by the ATM kinase (Karlseder et al. 1999; Takai et al. 2003). A major challenge in telomere biology is to define the mechanism by which functional telomeres prevent these events. Here we show that the human telomere-associated protein TRF2 is an inhibitor of the ATM kinase, suggesting a mechanism by which the telomeric protein complex prevents the activation of this DNA damage response transducer.

TRF2 is a small multimeric protein that binds to duplex telomeric (TTAGGG) repeats and recruits hRap1, ERCC1/XPF, WRN, and the Mre11/Rad50/Nbs1 complex to chromosome ends (Li et al. 2000; Zhu et al. 2000, Zhu et al. 2000 2003; Opresko et al. 2002; Machwe et al. 2004). TRF2 can be inhibited with a dominant-negative allele, TRF2^ΔBΔM^, which removes the endogenous TRF2 complex from telomeres (van Steensel et al. 1998). Upon expression of TRF2^ΔBΔM^, telomeres become uncapped and experience a series of deleterious events, including association with DNA damage response factors such as 53BP1, cleavage of the telomeric 3′ overhang by ERCC1/XPF, and telomere–telomere ligation by DNA ligase IV (van Steensel et al. 1998; de Lange 2002; Smogorzewska et al. 2002; Takai et al. 2003; Zhu et al. 2003). The DNA damage response to uncapped telomeres induces phosphorylation of DNA damage response proteins, including H2AX, SMC1, Rad17, CHK1, and CHK2, and upregulation of p53, p21, and p16, resulting in a G1 arrest (Karlseder et al. 1999; Smogorzewska and de Lange 2002; d'Adda di Fagagna et al. 2003). Primary human cells with telomere damage undergo apoptosis or senescence (Karlseder et al. 1999; Smogorzewska and de Lange 2002).

An important transducer of the DNA damage signal is the ATM kinase (reviewed in Shiloh 2003). ATM activation requires autophosphorylation on S1981 and concomitant dissociation into monomers, the presumed active form of the kinase (Bakkenist and Kastan 2003). DSBs and other genome stress lead to a rapid conversion of the ATM pool into active S1981–P monomers, which can phosphorylate regulators of the G1/S, intra-S, and G2/M cell cycle transitions (Bakkenist and Kastan 2003).

Activation of ATM also takes place in response to telomere damage. When telomeres become uncapped due to inhibition of TRF2, S1981-phosphorylated ATM associates with telomeres (Takai et al. 2003). Furthermore, ATM targets become phosphorylated in aging cells with shortened telomeres (d'Adda di Fagagna et al. 2003). Genetic evidence for a role of ATM in the telomere damage pathway is provided by the diminished ability of ataxia telangiectasia (A-T) cells to mount a DNA damage response after telomere uncapping (Karlseder et al. 1999; Takai et al. 2003). However, several lines of evidence suggest that a second PIKK (phosphatidylinositol 3-kinase-like kinase), such as ATR or DNA-PKcs, can transduce the telomere damage signal in the absence of ATM (Takai et al. 2003; Wong et al. 2003).

One proposed mechanism of telomere protection is based on the ciliate telomere proteins, which envelop the single-stranded telomere terminus (Horvath et al. 1998). Such a protein cap, if sufficiently stable, could simply hide chromosome ends from the DNA damage surveillance machinery. Both budding and fission yeast also contain protective single-stranded telomere-binding proteins, but it is not known whether these proteins function similarly by forming a physical cap over the telomere terminus (Garvik et al. 1995; Baumann and Cech 2001).

TRF2 must represent a different mechanism for telomere protection since it only binds to the duplex part of the telomere. TRF2 has been proposed to promote the formation of t-loops (Griffith et al. 1999; Stansel et al. 2001). In the t-loop configuration, the 3′ overhang of TTAGGG repeats is strand-invaded into the duplex part of the telomere. Although this could be an effective way to protect chromosome ends from nucleases and ligases, t-loops have several structural features resembling DNA lesions, including single strand to double strand transitions, 3′ and 5′ ends, and single-stranded DNA. Therefore, human telomeres may need additional mechanisms to circumvent checkpoint activation. The results presented here argue for a model in which TRF2 directly blocks activation of the ATM kinase.

## Results

**Figure 1:**
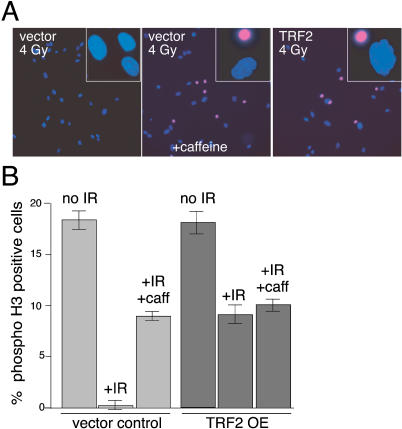
TRF2 Inhibits the IR-Induced Cell Cycle Arrest (A) Retrovirally infected IMR90 cells were treated with 4 Gy IR (left and right) or treated with 4 Gy IR and exposed to caffeine (10 mM) directly after irradiation (middle). After 16 h, during which the cells were incubated in 1 μg/ml colcemide, the DNA was stained with DAPI and mitotic cells were identified by immunofluorescence with an antibody to phosphorylated histone H3. (B) Quantification of bypass of IR-induced cell cycle arrest. The mean percentage of phosphorylated histone H3-positive cells and SDs from three experiments are given. The low maximal incidence of phosphorylated H3-positive nuclei (approximately 18%) is due to loss of mitotic cells during processing; loss of mitotic cells occurred at the same level in control and experimental samples.

TRF2 was overexpressed in IMR90 primary fibroblasts using a retroviral vector. Under these conditions, TRF2 saturates its telomeric binding sites and is present in the nucleoplasm. While control IMR90 cells showed the expected reduction in mitotic index after ionizing radiation (IR), TRF2 overexpression partially abrogated this checkpoint response, increasing the percentage of cells entering mitosis from 0.3% to 8% (Figure 1). The inappropriate entry into mitosis is indicative of a failure of the cell cycle checkpoints. Since cell cycle arrest after IR largely depends on ATM (reviewed in Shiloh 2003), we asked whether TRF2 was acting on this kinase. Caffeine, an inhibitor of ATM and the related kinase ATR, suppressed IR-induced arrest to a similar extent as TRF2 (Figure 1). Furthermore, caffeine had no additional effect on the ability of TRF2-overexpressing cells to bypass the DSB checkpoint, suggesting that TRF2 and caffeine target the same step in the response pathway.

**Figure 2:**
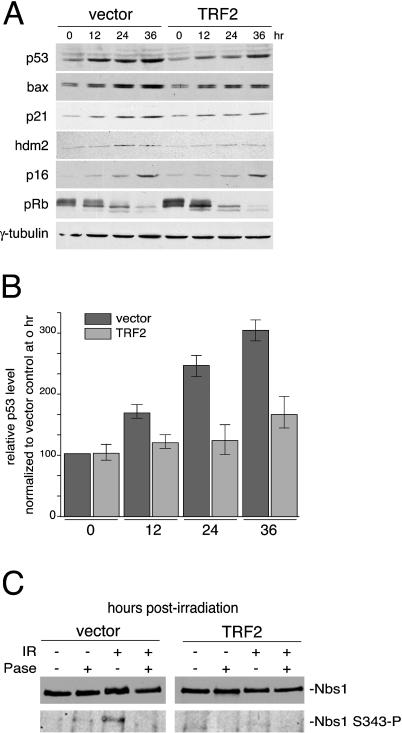
Effect of TRF2 on Downstream Readouts of the IR-Induced ATM Response (A) Retrovirally infected IMR90 cells were exposed to 5 Gy IR and harvested after 0, 12, 24, and 36 h. Levels of p53, Bax, p21, Hdm2, p16, pRB, and γ-tubulin (loading control) were detected by immunoblotting of equal cell number equivalents. (B) Amount of p53 protein at the indicated timepoints (hours) was determined by densitometry of p53 immunoblots such as shown in (A). Amounts were normalized to the vector control at 0 h. Mean values from three experiments and standard deviations are shown. (C) Retrovirally infected IMR90 cells were exposed to 20 Gy IR and harvested after 45 min. Nbs1 was immunoprecipitated and subsequently detected by immunoblotting using a general Nbs1 antibody and a phosphospecific Nbs1 S343 antibody. IPs were treated with λ-phosphatase where indicated.

We then asked whether TRF2 overexpression inhibited other ATM-dependent readouts of the DNA damage response. ATM phosphorylates and stabilizes p53 in response to DNA damage (reviewed in Kastan and Lim 2000). Quantitative immunoblotting showed that cells overexpressing TRF2 had a diminished ability to induce p53 after irradiation (Figures 2A and 2B). Both the relative level of p53 protein and the induction of its downstream targets p21, Bax, and Hdm2 were dampened. By contrast, the p16/Rb pathway was not affected by TRF2 (Figure 2A). We also examined the phosphorylation of Nbs1 on S343, a target of ATM (Gatei et al. 2000; Lim et al. 2000; Wu et al. 2000; Zhao et al. 2000). Phosphorylation of this residue causes a change in electrophoretic mobility shift and can also be detected using an antibody specific for the phosphorylated form of Nbs1. Extracts from irradiated control cells showed the previously reported retardation of Nbs1 and its reactivity with the S343–P-specific Nbs1 antibody (Figure 2C). Both alterations could be reversed by phosphatase treatment of the Nbs1 immunoprecipitations (IPs). In contrast, irradiation did not appear to induce phosphorylation of Nbs1 in cells overexpressing TRF2, indicating that TRF2 diminished the ATM-dependent phosphorylation of Nbs1 (Figure 2C).

**Figure 3:**
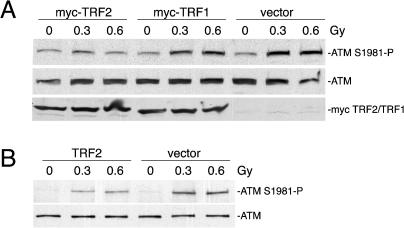
Effect of TRF2 on IR-Induced ATM Phosphorylation (A) Overexpression of TRF2 inhibits IR-induced phosphorylation of transfected ATM in 293T cells. 293T cells co-transfected with ATM and either TRF2, TRF1, or vector were treated with the indicated doses of IR. After a 30-min recovery, cells were harvested and immunoblot analysis was performed on whole-cell lysates. (B) Overexpression of TRF2 inhibits IR-induced phosphorylation of endogenous ATM in primary fibroblasts. IMR90 primary fibroblasts infected with a retroviral construct expressing TRF2 or an empty virus were treated with the indicated doses of IR. After a 1 h recovery, cells were harvested and ATM was immunoprecipitated from whole-cell lysates. Immunoblot analysis was performed on immunoprecipitated ATM.

Because TRF2 overexpression blunted several cellular responses that depend (in part) on the ATM kinase, we determined TRF2's effect on the activation of ATM itself. Phosphorylation of ATM on S1981, an early and essential step in the activation of this kinase, can be detected rapidly after IR, even when a low level of DNA damage is induced (Bakkenist and Kastan 2003). To test whether TRF2 affected the autophosphorylation of ATM, the two proteins were expressed in 293T cells and ATM was activated with low doses of IR. TRF2 inhibited the activation of ATM as monitored by immunoblotting with an antibody specific for ATM S1981–P (Figure 3A). The relative level of ATM S1981–P (normalized to total ATM protein) at 0.3 Gy was 49% of the vector control value (p = 0.002, Student's t test; n = 7). The TRF2 paralog TRF1, which also binds the duplex telomeric repeats, did not have a significant effect on ATM activation, demonstrating that the effect on ATM is specific to TRF2 (Figure 3A). Overexpression of TRF2 also diminished the IR-induced ATM autophosphorylation of endogenous ATM in IMR90 fibroblasts to 55% of vector control value at 0.3 Gy and 60% of vector control value at 0.6 Gy (Figure 3B).

**Figure 4:**
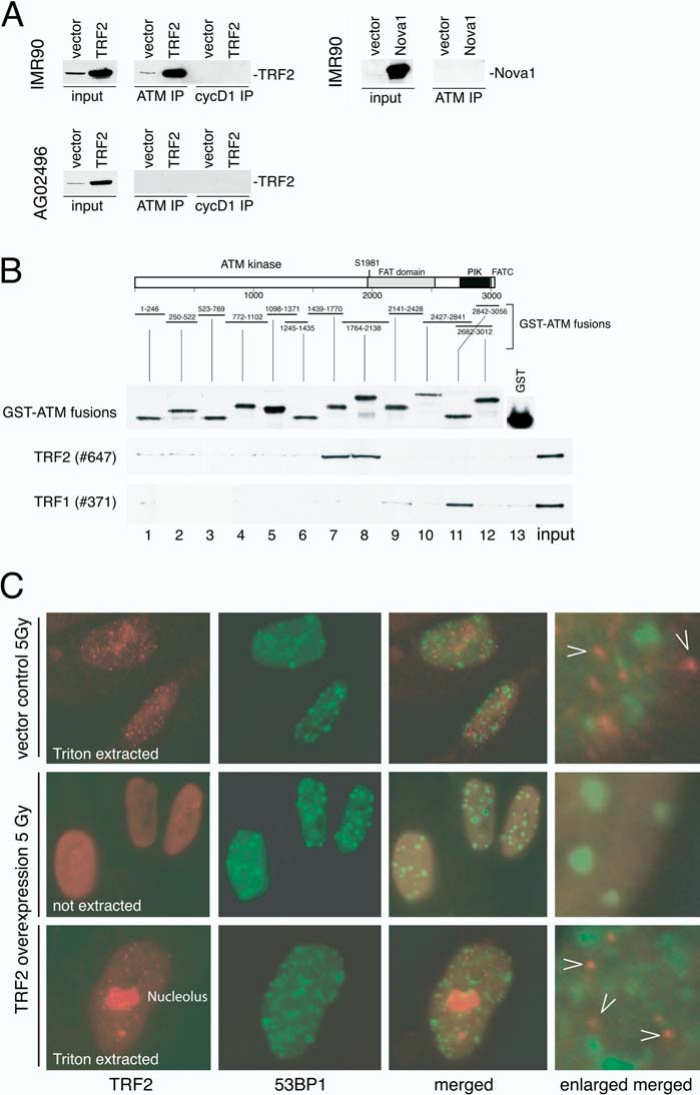
TRF2 Interacts with the ATM Kinase In Vivo and In Vitro and TRF2 Does Not Localize to IRIF (A) Co-IP of TRF2 with ATM. Protein extracts from IMR90 cells and A-T cells (AG02496) infected with an empty virus or a TRF2-overexpressing virus were incubated with anti-ATM or anti-Cyclin D1 antibodies as indicated, and TRF2 was detected in the IP pellets by immunoblotting. The right panel represents IPs with anti-ATM antibodies from IMR90 cells infected with a retrovirus overexpressing Nova1 or the empty vector and detection of Nova1 by immunoblotting. For each extract 1% of the IP input (input) was processed for immunoblotting in parallel. (B) Bacterially expressed ATM–GST fusion proteins were purified on glutathione agarose beads and visualized by Western blotting with anti-GST antibody (Upstate Biotechnology [Lake Placid, New York, United States] #06–332) (top). Unfused GST was run on a separate gel because of its low molecular weight. Equal amounts of fusion proteins and GST alone were incubated with purified baculoviral TRF2 (middle) or TRF1 (bottom), bound to glutathione beads, spun down, washed, and bound proteins were processed for immunoblotting with an anti-TRF1 or anti-TRF2 serum. (C) TRF2 does not localize to IRIFS. IMR90 primary fibroblasts infected with a retroviral construct expressing TRF2 or an empty virus were treated with 5 Gy IR. After a 90 min recovery, cells were fixed and processed for immunofluorescence with or without Triton X-100 extraction before fixation. Arrowheads denote foci of TRF2 signal previously demonstrated to represent telomeres. When overexpressed, some TRF2 is localized to nucleolus.

In order to understand the mechanism by which TRF2 inhibited ATM, we determined whether they interacted in vivo. IPs of the ATM kinase from primary human IMR90 fibroblasts resulted in recovery of a small fraction (approximately 1%) of endogenous TRF2 (Figure 4A). This association was accentuated when TRF2 was overexpressed from a retroviral vector. TRF2 was not recovered in anti-ATM immunoprecipitates from A-T cells even when TRF2 was overexpressed (Figure 4A), demonstrating that the recovery of TRF2 is dependent on the presence of functional ATM. The co-IP of TRF2 with ATM from IMR90 cells was resistant to the addition of ethidium bromide (data not shown), arguing that DNA tethering is not responsible for the association. A control IP with antibodies to the CycD1/Cdk4/Cdk6 kinase complex did not precipitate TRF2, and an irrelevant nuclear protein (Nova1), overexpressed in parallel in IMR90 cells, was not recovered in the ATM IP (Figure 4A). The association of TRF2 with ATM was not dependent on the presence of DNA damage, since neither IR nor UV treatment enhanced the recovery of TRF2 in ATM IPs (data not shown).

The nature of the association of TRF2 with ATM was explored further using in vitro pulldown experiments. GST-tagged fragments of ATM were tested for their ability to bind purified TRF2 protein expressed in a baculovirus system. In parallel, we used baculovirus-derived TRF1, which was previously shown to interact with ATM by co-IP (Kishi et al. 2001). TRF2 bound to two overlapping fragments of ATM spanning amino acids 1439 to 2138 (Figure 4B). This region contains the FAT domain and S1981, the critical target of autophosphorylation (Bakkenist and Kastan 2003). TRF1 bound a different region of ATM (Figure 4B), demonstrating the specificity of the observed interactions.

Blunting of the DNA damage response was observed when TRF2 was overexpressed throughout the nucleus. Because TRF2 is chiefly present at telomeres, the simplest interpretation is that the observed activity reflected a telomeric function. However, we also considered the possibility that TRF2 may have a heretofore clandestine role in the general DNA damage response. If this were true, TRF2 might be expected to localize to IR-induced foci (IRIF), where it would be in a position to modulate ATM. Previous data had shown that the endogenous TRF2 does not relocate from telomeres to IRIF (Zhu et al. 2000). Similarly, immunofluorescence analysis showed that overexpressed TRF2 did not form IRIF (Figure 4C): the pattern of TRF2 localization was unchanged by IR, and there was no detectable colocalization with the known IRIF component 53BP1 (Schultz et al. 2000). This was also the case when TRF2 localization was examined in cells from which the nucleoplasmic proteins were extracted with a mild detergent (Figure 4C). Thus, the inhibitory effect of TRF2 on ATM signaling does not reflect association of TRF2 with sites of DNA damage. Instead, we propose that the inhibition of ATM by TRF2 is an innate property of the protein, important at its natural location: telomeres. However, we cannot exclude the transient presence of TRF2 at DNA lesions and/or a role for TRF2 in the general DNA damage response.

## Discussion

Natural chromosome ends require mechanisms to prevent the activation of the DNA damage response. Inhibition of the ATM kinase at human telomeres is particularly important since the telomeric complex contains the Mre11 complex, one of the DNA damage sensors of the ATM pathway (Carson et al. 2003; Petrini and Stracker 2003; Uziel et al. 2003). The telomeric protein TRF2 appears to play a central role in preventing telomeres from activating ATM. Removal of TRF2 from telomeres results in the localization of the active, phosphorylated form of ATM to unprotected chromosome ends (Takai et al. 2003) and induces ATM-dependent apoptosis (Karlseder et al. 1999). The data reported here are consistent with the hypothesis that TRF2 protects telomeres through a direct interaction with ATM that blocks its activation. As a result, TRF2 abrogates the downstream outcomes of the ATM-dependent DNA damage response, including phosphorylation of various ATM targets and cell cycle arrest.

We feel that the interaction of TRF2 with ATM is likely to be relevant to the mechanism by which TRF2 blocks ATM signaling. TRF2 binds ATM in a region surrounding S1981, which is functionally linked to the oligomerization state of ATM. Phosphorylation on S1981 occurs concomitant with the dissociation of ATM dimers (or oligomers), forming the monomeric, active form of the kinase (Bakkenist and Kastan 2003). TRF2 is also an oligomer of four to eight subunits, which are held together by the TRFH dimerization domain as well as other, yet to be defined, protein interactions (Broccoli et al. 1997; Fairall et al. 2001; Stansel et al. 2001). Owing to its oligomeric nature, TRF2 could potentially cross-link ATM monomers and thus hold the kinase in its inactive dimeric (or oligomeric) state. In this manner, TRF2 could abrogate the ATM pathway since it would block amplification of the ATM signal at an early step. In agreement with this idea, the in vitro GST pulldown experiments showed that TRF2 can interact with ATM when it is not phosphorylated on S1981. Because mutations in the TRF2 dimerization domain destabilize the protein, it has not been possible to test the contribution of TRF2 oligomerization on ATM repression directly. Alternatively, the interaction of TRF2 with the region surrounding S1981 may prevent ATM autophosphorylation or TRF2 binding could block a presumed interaction between ATM and a DNA damage sensor, such as the Mre11 complex. It is unlikely that TRF2 acts as an ATM target mimetic that titrates out genuine ATM targets since TRF2 is not a target of the ATM kinase (R. Drissi, M. B. Kastan, and J. Dome, unpublished data). Furthermore, TRF2 does not block ATM kinase activity in an in vitro assay (S. Kozlov, J. Karlseder, and M. F. Lavin, unpublished data). These findings are consistent with TRF2 acting at one of the earlier steps in the activation of the ATM kinase, including the interplay between ATM and DNA damage sensors, ATM autophosphorylation, or dissociation of ATM dimers.

In this study we have expressed TRF2 at high levels throughout the nucleus, whereas endogenous TRF2 is localized primarily to telomeres (van Steensel et al. 1998). Our estimates suggest that TRF2 is extremely abundant at telomeres. Human cells contain on the order of 1 million copies of TRF2 (X.-D. Zhu and T. de Lange, unpublished data), sufficient to position thousands of TRF2 molecules at each chromosome end. This number is consistent with the presence of thousands of TRF2-binding sites per telomere and the oligomerization potential of the protein. Thus, for every ATM kinase that could be activated at a chromosome end, there is a vast molar excess of its potential inhibitor, TRF2. Since TRF2 is specifically lodged at telomeres and remains there when DNA damage is induced, it is unlikely to interfere with activation of the ATM kinase at sites of DNA damage elsewhere in the genome. Hence, TRF2 could act as a telomere-specific inhibitor of ATM.

Previous studies have shown that overexpression of TRF2 can protect critically short telomeres generated by replicative aging (Karlseder et al. 2002). TRF2 reduced the incidence of end-to-end chromosome fusions in this setting and also delayed the onset of senescence. These findings suggested that senescence is induced by an altered telomere state, in which the telomere has become so short that the amount of TRF2 it can recruit is insufficient for the protection of the chromosome end. When TRF2 is overexpressed, this deficiency in TRF2 recruitment may be overcome. One possibility is that the altered state of critically short telomeres represents a situation in which telomeres have a diminished ability to form t-loops. The current findings raise the possibility that the altered state may also include a situation in which the telomere contains insufficient TRF2 to repress ATM. However, the ability of TRF2 to delay senescence was also observed in A-T cells (Karlseder et al. 2002), indicating that ATM repression is not the only pathway by which increased TRF2 loading can protect critically short telomeres.

As TRF2 can bind ATM, it has the inherent ability to recruit this protein to telomeres. ATM has not been observed at undamaged telomeres, but its abundance may be too low for detection. The idea that ATM could be recruited to telomeres by TRF2 is interesting considering that ATM-like kinases are necessary for telomere maintenance in Saccharomyces cerevisiae (Craven et al. 2002) and Schizosaccharomyces pombe (Matsuura et al. 1999). It is not excluded that human telomere maintenance similarly requires ATM. TRF2 could function to recruit ATM in an inactive form, perhaps allowing for highly regulated activation of ATM at appropriate times. Such regulation of DNA damage signaling and repair pathways at telomeres has been proposed previously in the context of the nonhomologous end-joining pathway and the role of the nucleotide excision repair endonuclease ERCC1/XPF (Smogorzewska et al. 2002; Zhu et al. 2003). In both cases, proteins with the potential to have detrimental effects on telomeres appear to be regulated such that their activities can be employed for telomere function.

## Materials and Methods

### 

#### Cell culture and IR treatment

IMR90 primary lung fibroblasts (ATCC, Manassas, Virginia, United States) and AG02496 and AG04405 primary A-T fibroblasts (Coriell Cell Repository, Camden, New Jersey, United States; PD 12 and PD 15) were grown and infected with retroviruses as described elsewhere (Karlseder et al. 2002). For γ-irradiation, 3 10^5^ cells were seeded in 5-cm culture dishes and exposed to a Ce_137_ source. Where indicated, the medium was replaced with medium containing 10 mM caffeine (Sigma, St. Louis, Missouri, United States) and 1 μg/ml colcemide (Sigma). Indirect immunofluorescence was performed as described (Smogorzewska et al. 2000; Takai et al. 2003).

#### Co-transfection assay for S1981–P inhibition

One day prior to transfection, approximately 5 10^6^ 293T cells were plated in 10-cm dishes. Cells were transfected with 1 μg of FLAG–ATM DNA (Canman et al. 1998) and 9 μg of N-terminally Myc-tagged TRF2 (Karlseder et al. 2002) or TRF1 in a pLPC vector backbone (gift of S. Lowe, Cold Spring Harbor Laboratory) or vector alone using CaPO_4_ coprecipitation. Two days after transfection, cells were harvested in media and divided into three equal fractions, which were exposed to 0, 0.3, or 0.6 Gy IR. Cells were allowed to recover for 30 min, washed with PBS, and resuspended in 250 μl of lysis buffer (50 mM Tris [pH 7.4], 1% Triton X-100, 0.1% SDS, 150 mM NaCl, 1 mM EDTA, 1 mM DTT, 1 mM PMSF, with a complete mini-protease inhibitor tablet [Roche, Basel, Switzerland] per 10 ml). The NaCl concentration was raised to 400 mM, and the lysate was incubated on ice for 5 min. The NaCl concentration was reduced to 200 mM, cell debris was removed by centrifugation, and an equal volume of Laemmli buffer was added to the lysate.

#### Immunoblotting

For ATM immunoblots in the ATM S1981–P suppression assays: 40 μl of 293T cell lysate or ATM immunoprecipitated from approximately 5 10^6^ IMR90 fibroblasts were run on 7.5% precast polyacrylamide Bio-Rad (Hercules, California, UnitedStates) Ready Gels. PVDF Immobilon^TM^ Transfer Membrane (Millipore, Billerica, Massachusetts, United States) was prepared for protein transfer according to the manufacturer's instructions and the gel was transferred for 2 h at 90 V. Membranes were preincubated in 10% milk, 0.1% Tween-20 in PBS for 30 min at room temperature and subsequently incubated with primary antibodies: polyclonal rabbit ATM S1981–P (Bakkenist and Kastan 2003) and mouse monoclonal ATM antibody MAT3 (gift from Y. Shiloh) diluted in 0.1% milk, 0.1% Tween-20 in PBS overnight at 4 °C followed by three 10 min washes. Membranes were incubated for 45 min with HRP-conjugated secondary antibodies, washed, and developed using the ECL system (Amersham, Little Chalfort, United Kingdom). Immunoblots of Myc-tagged proteins (using Ab-1; Oncogene Research, Cambridge, Massachusetts, United States) in the ATM S1981–P suppression assays were performed as above, except that nitrocellulose (Schleicher and Schuell, Keene, New Hampshire, United States) filters were used. For all other immunoblots, cells were trypsinized, washed once with PBS, and subsequently lysed in Laemmli buffer at 10^4^ cells/μl. Lysates (10 μl) were separated on SDS-polyacrylamide gels (29:1 acrylamide: bisacrylamide, 8% for p53, TRF2, and γ-tubulin, 6% for pRB and Hdm2, 12% for Bax, p21, and p16) and transferred onto nitrocellulose membranes (Schleicher and Schuell) for 60 min at 90 V (Bio-Rad Mini-Protean II Cell). Membranes were preincubated in 10% nonfat dry milk, 0.1% Tween-20 in PBS for 30 min and subsequently incubated with primary antibodies: p53 D01 (Santa Cruz Biotechnology, Santa Cruz, California, United States); TRF2 serum 647 (Zhu et al. 2000); pRB #554136 (PharMingen, Uppsalla, Sweden); Hdm2 #3F3 (gift from, A. Levine); Bax #sc-7480 (Santa Cruz Biotechnology); p21 sc-7480 (Santa Cruz Biotechnology); p16 #15126E (PharMingen); γ-tubulin GTU88 (Sigma); Nova1 (Luque et al. 1991) in 5% dry milk, 0.1% Tween-20 in PBS overnight. Secondary antibody incubation and ECL were performed as described above. To quantify signals, band intensities were determined using an AlphaImager^TM^ 2200 using the SpotDenso function of AlphaEaseFC^TM^ Software Version 3.1.2 (Witec, Littau, Switzerland).

#### IP

For co-IP of ATM and TRF2, proteins were extracted from subconfluent cells by incubating trypsinized cells (approximately 10^7^ cells/0.1 ml buffer) in 20 mM HEPES (pH 7.9), 0.42 M KCl, 25% glycerol, 0.1 mM EDTA, 5 mM MgCl_2_, 0.2% NP40, 1 mM DTT, 0.5 mM PMSF, 1 μg/ml leupeptine, 1 μg/ml aprotinin, 10 μg/ml pepstatin on ice for 30 min. Debris was removed by centrifugation (14,000 rpm, 4 °C, 10 min). Protein concentration in the supernatant was determined using the Bradford assay and 400 μg of protein was diluted to 150 mM KCl and incubated for 20 min with 100 μl of protein G–Sepharose beads (Amersham), blocked with 1% fetal bovine serum in PBS. The beads were collected at 14,000 rpm for 1 min and the supernatant was incubated with 5 μg of anti-ATM antibody (AB3, Oncogene Research) or anti-Cyclin D1 antibody (#sc-6281, Santa Cruz Biotechnology) for 1 h at 4 °C on a nutator. Protein G–Sepharose beads (30 μl) were added, and the mixture was incubated 1 h at 4 °C on a nutator. The beads were collected at 4,000 rpm at 4 °C, washed three times with wash buffer (150 mM NaCl, 1% NP40, 50 mM Tris [pH 8.0] with protease inhibitors as described above) by vortexing the suspension for 10 s. The beads were resuspended in Laemmli buffer and boiled 5 min, and proteins were separated by polyacrylamide gel electrophoresis. For IP of endogenous ATM from IMR90 fibroblasts for the phosphorylation assay, cells were resuspended in lysis buffer (50 mM Hepes [pH 7.5], 150 mM NaCl, 50 mM NaF, 1% Tween-20, 0.2% NP40, 1mM PMSF, with 1 complete mini-protease inhibitor tablet [Roche, Basel, Switzerland] ) and centrifuged at 13,000 rpm for 10 min. 400 μl of lysate was incubated with 30 μl of blocked protein G beads and 100 μl of D16.11 monoclonal supernatant (Alligood et al. 2000) for 1.5 h. Beads were washed once in lysis buffer and twice in RIPA buffer and resuspended in 60 μl of Laemmli buffer.

#### Pulldown assays

GST–ATM fusion plasmids (Khanna et al. 1998) were transformed into BL-21 cells. A 10-ml overnight culture was used to inoculate 500 ml of LB-Amp (50 μg/ml) and at OD_600_, 0.5–0.7, 0.3 mM IPTG (final) was added. After 3 h at 30 °C, cells were harvested, resuspended in 8 ml of lysis buffer (50 mM Tris [pH 7.9], 100 mM KCl, 1% Triton X-100, 2 mM DTT, 0.1 mM PMSF, 1 complete protease inhibitor tablet [Roche]) and sonicated three times for 30 s on ice. The lysate was cleared by centrifugation at 50,000 g at 4 °C and incubated with 600 μl of equilibrated glutathione beads for 2 h at 4 °C. The beads were washed three times for 10 min each (washes 1 and 3: PBS, 1% Triton X-100, 2 mM DTT, 0.1 mM PMSF, 1 mM benzamidine, 1 complete protease inhibitor tablet [Roche]; wash 2: 300 mM NaCl, 50 mM Tris [pH 7.9], 2 mM DTT, 0.1 mM PMSF, 1 mM benzamidine, 1 complete protease inhibitor tablet [Roche]) and a fourth time in wash 4 (50 mM Tris [pH 7.9], 100 mM KCl, 10% glycerol, 2 mM DTT, 0.1 mM PMSF). Fusion proteins were eluted in 500 μl of wash 4 containing 15 mM glutathione (reduced form). Two subsequent elutions were collected. Five micrograms of GST fusion proteins or GST alone were incubated with 2 μg of baculoviral TRF1 or TRF2 in binding buffer (150 mM NaCl, 100 mM KCl, 50 mM Tris [pH 8.0], 1% NP40, 0.1% SDS, 100 g/ml BSA) at 4 °C for 1 h. Glutathione beads (20 μl) were added and incubated for 1 h at 4 °C. Beads were collected by centrifugation at 5,000 rpm at 4 °C and washed three times for 10 min each with binding buffer, and bound protein was eluted by boiling the samples in Laemmli buffer. GST fusion proteins, TRF1, and TRF2 were detected by immunoblotting.

#### Cell cycle arrest assay

Cells were seeded on microscope coverslips and irradiated with 4 Gy of γ-irradiation as described above. Cells were incubated in growth medium containing 1 μg/ml colcemid for 16 h. Cells were fixed, and phosphorylated histone H3 was detected by indirect immunofluorescence using a phosphospecific antibody (6G3 monoclonal; Cell Signaling Technology, Beverly, Massachusetts, United States). Cells in mitosis were counted and expressed as a percentage of total cell number.

## Abbreviations

A-T: ataxia telangiectasia
DSB: double-stranded break
IP: immunoprecipitation
IR: ionizing radiation
IRIF: ionizing radiation-induced foci
